# Characterization of Vertically Aligned Carbon Nanotube Forests Grown on Stainless Steel Surfaces

**DOI:** 10.3390/nano9030444

**Published:** 2019-03-15

**Authors:** Eleftheria Roumeli, Marianna Diamantopoulou, Marc Serra-Garcia, Paul Johanns, Giulio Parcianello, Chiara Daraio

**Affiliations:** 1 Department of Mechanical and Process Engineering, Swiss Federal Institute of Technology (ETH Zurich), 8092 Zurich, Switzerland; eroumeli@caltech.edu (E.R.); mdiamant@student.ethz.ch (M.D.); marc.serra.g@gmail.com (M.S.-G.); paul.johanns@epfl.ch (P.J.); 2 Division of Engineering and Applied Science, California Institute of Technology, Pasadena, CA 91125, USA; 3 Department of Physics, Swiss Federal Institute of Technology (ETH Zurich), 8092 Zurich, Switzerland; 4 General Electric Switzerland, CH-5401 Baden, Switzerland; giulio.parcianello@gmail.com

**Keywords:** carbon nanotube, steel, characterization, mechanical properties, electron microscopy, coatings

## Abstract

Vertically aligned carbon nanotube (CNT) forests are a particularly interesting class of nanomaterials, because they combine multifunctional properties, such as high energy absorption, compressive strength, recoverability, and super-hydrophobicity with light weight. These characteristics make them suitable for application as coating, protective layers, and antifouling substrates for metallic pipelines and blades. Direct growth of CNT forests on metals offers the possibility of transferring the tunable CNT functionalities directly onto the desired substrates. Here, we focus on characterizing the structure and mechanical properties, as well as wettability and adhesion, of CNT forests grown on different types of stainless steel. We investigate the correlations between composition and morphology of the steel substrates with the micro-structure of the CNTs and reveal how the latter ultimately controls the mechanical and wetting properties of the CNT forest. Additionally, we study the influence of substrate morphology on the adhesion of CNTs to their substrate. We highlight that the same structure-property relationships govern the mechanical performance of CNT forests grown on steels and on Si.

## 1. Introduction

The unique properties of carbon nanotube (CNT) forests have been documented extensively in the past decade [1,2,3,4]. Modifying the synthesis conditions allows tuning of the geometry, alignment, density, and structure of CNTs, which offers control over their mechanical performance [5,6,7,8,9,10]. CNT forests grown directly on metal substrates, and specifically on steels, are particularly interesting because they offer a conformal coating solution, which is independent from the substrate’s geometry [11,12,13,14,15,16,17,18,19,20,21,22,23,24,25,26,27,28,29,30,31,32,33,34,35,36]. Direct growth avoids the need for intermediate adhesive layers, thus enabling a robust contact interface between the CNTs and the metal [12].

Floating catalyst chemical vapor deposition combines high CNT yield, vertical alignment, and conformal substrate coverage. This method is commonly used for CNT forest growth on metal substrates [12,15,25,34]. Most literature on CNTs grown on metallic substrates, either in the form of aligned forests or non-aligned CNTs, focuses on the synthesis methods [11,15,18,19,20,21,22,23,24,25,37]. Few reports characterize the super-hydrophobicity of CNTs [33,34,35], their corrosion resistance [33,38], field-emission properties [27,32], and electrochemical performance as capacitors [12,26,28]. The mechanical response of CNT forests grown on metallic substrates, like their behavior under compression or impact, as well as their adhesion to the substrate, have not yet been thoroughly investigated.

The remarkable compressive behavior of CNT forests grown on silicon has been documented in literature and includes energy dissipation in the order of MJ/m [25,39,40], compressive strengths in the range of 0.1–500 MPa [9,39], and almost full-length strain recovery (>80%) for hundreds of compression cycles [1,41]. The mechanical performance of silicon-grown CNT forests has been correlated to the forests’ structure and alignment [6,7,8,9,41]. The forests’ structural properties, in turn, depend on the catalyst distribution and interfacial effects between the CNTs and their substrate [9,42,43]. However, the behavior of catalysts at the interfaces with the steel substrates is different, due to the native composition of these substrates. Steels, for example, are rich in multiple metals and oxides. In the floating catalyst method, the interactions of the supplied metal catalyst particles with the native metals and oxides of the growth substrate determine the rate and yield of CNT formation [15]. We expect the differences in surface composition between silicon and steel substrates to affect the morphology and ultimate mechanical behavior of the CNT forests grown on the different types of substrates. The focus of this work is to study the mechanical properties, adhesion, and wettability of CNT forests grown on steel substrates and reveal the structure-property relationships that govern CNT performance. Our results demonstrate the mechanical performance of CNT forests grown on steel is governed by the CNT micro-structure in the same way as it does on Si-grown CNTs.

Considering the fact that the CNT forests combine light-weight, super-hydrophobicity, high energy absorption, similar to that of rigid polymer foams, with reusability, due to the large strain recoverability, a wide area of steel coating applications can be reached.

## 2. Materials and Methods

The compositions of the used steels are summarized in Table 1 (samples named S1–S4 as designated in Table 1). Prior to growth, all the substrates were placed in a Cr-etching bath for 15 min and then ultra-sonicated in acetone for 5 min to remove any organic residues or dust. In addition, the substrates were air-annealed for 30 min at 827 °C before the start of the growth process. The surface roughness of all substrates was measured with a Dektak XT Stylus profilometer (Bruker, Billerica, MA, USA) and the values are summarized in Table 1.

The steels’ surface energy was determined through contact angle measurements using a Drop Shape Analyzer (DSA25, Krüss Optronic GmbH, Hamburg, Germany) [44]. Young’s theory relates the measured contact angle (*θ*) to the specific free energy of the tested solid (γ_S_), the used liquid (γ_L_), and the interfacial free energy between the solid and the liquid (γ_SL_) as follows [44]:

(1) $$\gamma_{L}cos\theta =\gamma_{S}-\gamma_{SL}.$$

Fowkes’ theory predicts the surface energy of a solid, assuming it is a summation of individual and independent components (polar, dispersive, acidic, etc.):

(2) $$\gamma_{S}=\gamma_{s}^{p}+\gamma_{s}^{d}+\gamma_{s}^{ab}+\ldots ,$$

where the superscripts “*d*”, “*p*”, and “*ab*” correspond to the dispersive, polar, and acid-base interactions respectively.

We used water and diiodomethane to probe the polar and dispersive forces of the substrates and a harmonic-mean approach to sum their contribution following Wu’s equation [44]:

(3) $$\left(1+cos\theta \right)\gamma_{L}=4\left(\frac{\gamma_{L}^{d}\gamma_{S}^{d}}{\gamma_{L}^{d}+\gamma_{S}^{d}}+\frac{\gamma_{L}^{p}\gamma_{S}^{p}}{\gamma_{L}^{p}+\gamma_{S}^{p}}\right),$$

where the subscript “*L*” is for each liquid used in the measurement.

CNT forests were synthesized on thermally oxidized silicon (Si) wafers and/or on the different stainless steel substrates, using vapor phase or “floating catalyst” thermal chemical vapor deposition (CVD). The furnace tube had an external diameter of 5 cm and a 15 cm heating zone [10]. The synthesis reactions were performed in Ar flow of 800 sccm at 827 °C in atmospheric pressure. The precursor solution was ferrocene in toluene, at a concentration of 0.02 g/mL and the injection rate was 1 mL/min.

**Figure 1:**
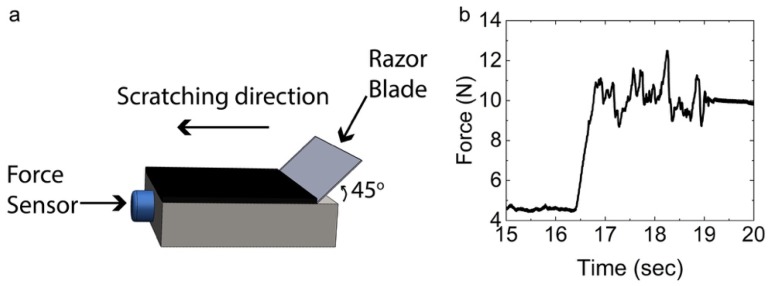
(**a**) Schematic of the scratching setup for testing the adhesion between the CNTs and their steel substrates; (**b**) obtained force-time curve for sample S3.

To characterize the CNT structure and purity we used scanning electron microscopy (SEM) and Raman spectroscopy. SEM images were obtained using a SU8200 SEM (Hitachi, Tokyo, Japan), operating at 2–5 kV and 11 nA (Figure 1). High resolution transmission electron microscopy (HRTEM) images were obtained using a TF30ST (FEI, Hillsboro, OR, USA) operating at 300 kV.

Following the method by Laurent et al. [45], the mass and volume of a single multi-walled CNT, *m_MWCNT_* and volume *V_MWCNT_*, can be calculated as follows:

(4) $$m_{\mathrm{MWCNT}}=\frac{1}{A}\pi L\left[nd_{out}-2d_{s-s}\sum_{i=0}^{n}i\right],$$

(5) $$V_{MWCNT}=\frac{\pi Ld_{out}^{2}}{4}.$$

In Equation (4), *A* is the specific surface area of a single nanotube sheet (equivalent to one side of a rolled graphene sheet regardless of its diameter, *A* = 1315 m^2^/g), *L* is the CNT length, *n* is the number of walls of the CNT, *d_out_* is the outer diameter, *d*_*s*−*s*_ is the inter-tube distance (*d*_*s*−*s*_ = 0.34 nm).

Raman spectroscopy measurements were performed on a NTEGRA Spectra (NT-MDT SI LLC, Moscow, Russia). The hydrophobicity response of the CNT forest was determined through the water contact angle measurements described in the previous section.

The micromechanical testing of the samples was performed using a FT-MTA02 (FemtoTools AG, Zurich, Switzerland). The obtained data of this experiment also include contributions from the sensor and system configuration as well as from the supporting steel material underneath the CNT forest. In order to remove these contributions and allow further calculations on the response of the CNT forests, we also tested a polished uncoated SS substrate [46]. At least four experiments for every sample type were performed, and the average values for dissipated energy, unloading modulus (*E*), and peak stress are extracted from the obtained stress-strain curves.

The samples’ recovery, *R*, is defined as the displacement recovered at the end of each compression cycle, divided by the maximum displacement, according to the following formula [10]:

(6) $$R=\frac{\varepsilon_{max}-\varepsilon_{unload}}{\varepsilon_{max}},$$

where *ε_max_* is the maximum displacement at the end of the compression cycle, and *ε_unload_* is the displacement after unloading to 10% of the maximum load in each cycle.

The loss coefficient, *η*, measures energy dissipation and is calculated as follows [47]:

(7) $$\eta =\frac{\Delta U_{i}}{2\pi U_{1}},$$

where *U_1_* is the elastic energy stored in the material when it is loaded elastically to a stress *σ_max_* during the first cycle, and *ΔU_i_* is the energy dissipated in the *i*th cycle.

Several methods have been reported to study CNT adhesion to metal substrates including peel tests, scotch tape tests, scratch tests, indentation, and combinations of these [48,49]. Amongst these, the scratch test is the most widely accepted method to quantitatively assess well-adherent coatings on metals [48,50,51]. To specifically study the adhesion of CNTs to their growth substrates, the methods reported in the literature include: the scotch tape removal method [33,34], the pull-off method, which involves taping over the CNTs and measuring the pulling force [12,14], and the ultrasonication method, in which the time required to remove the CNTs from the substrate is recorded [17,33]. However, these methods are often user-sensitive, uncalibrated, and do not guarantee that the CNTs will be removed from the substrate as opposed to breaking at weak points [52]. More accurate methods proposed for adhesion testing employ a nano-indenter or an atomic force microscope (AFM) [52,53], which scratches through the substrates and measures the coating adhesion strength. Nevertheless, these approaches are limited by a low force detection threshold, and the dimensions of the coating may impose additional restrictions. Thus, in order to study the adhesion of CNT forests to their growth substrates, we develop a custom-built scratch setup.

A schematic of the custom scratch setup is presented in Figure 1a. The setup consists of a 3-axis linear stage holding a razor blade (No.9, VWR International, Radnor, PA, USA) adjusted at a 45° angle above the substrate. A strain gauge is placed in the x-direction (scratching direction), to detect the force on the substrate while the razor blade scratches off the CNT forest. After positioning the sample on the stage using adjustment screws, the razor blade is moved manually, so that the blade is in contact with the edge of the CNT forest. The samples are scratched with five different scratching speeds, namely 0.2, 0.5, 1.0, 2.0, and 5.0 mm/s. The output voltage of the strain gauge is recorded as a function of time with an oscilloscope (DP0314, Tektronix Inc., Beaverton, OR, USA). After calibration (*K_c_* = 0.045 V/N), the scratching force can be obtained. A representative example of an obtained force-time curve, corresponding to sample S3 is presented in Figure 1b.

From the force, the energy release per unit area (E/A, in J/m^2^) can be calculated as follows:

(8) $$\frac{E}{A}=\frac{E}{w\times L}=\frac{E}{w\times u\times t}=\frac{P}{w\times u}=\frac{F\times u}{w\times u}=\frac{F}{w},$$

where *w* is the width of the CNT forest, *P* is the power in J/s, *L* is the length of the forest, and *u* is the velocity in m/s.

## 3. Results

### 3.1. Structural Investigation

**Figure 2:**
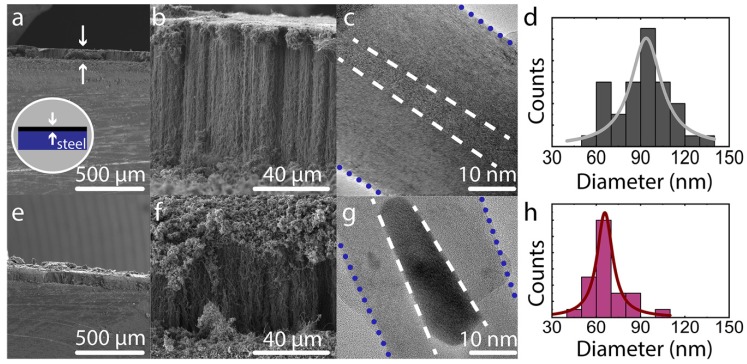
(**a**,**b**) Representative SEM images of sample S1; (**c**) HRTEM image of individual CNT from sample S1; dash lines mark the inner tube wall and dotted lines mark the outer CNT wall; (**d**) outer diameter size distribution for sample S1; (**e**,**f**) SEM images of sample S4; (**g**) HRTEM image of individual CNT from sample S4; (**h**) measured outer diameter size distribution for sample S4.

In Figure 2, we present typical SEM and HRTEM images of CNTs forests grown on the S1 and S4 substrates. We observe a conformal coating of about 60–80 µm in every case on the top of the steel surface. The average growth rate is the same across all samples grown on steels, 1.4 ± 0.2 µm/min. The average outer nanotube diameter, as calculated from SEM images, is 93 ± 20 nm for CNTs grown on S1–S2 and 65 ± 20 nm for CNTs grown on S3–S4. HRTEM images reveal that CNTs grown on S1–S2 have on average 107 walls, while those on S3–S4 have on average 68 walls. The small height of the grown CNT forests does not allow accurate direct mass measurement. To calculate their mass, we employ the method suggested by Laurent et al. [45] as mentioned in the previous section. From the SEM-observed areal density (7.9 × 10^8^ CNTs/cm^2^ for S1–S2 and 1.4 × 10^9^ CNTs/cm^2^ for S3–S4) and the overall forest volume (area of steel substrate x forest height), we calculate the sample density. Based on HRTEM and SEM observations, this method results on a density of 0.1 g/cm^3^ for samples S1–S2, and 0.06 g/cm^3^ for S3–S4, which are within the literature-reported values for CNT forests [6,10,54]. Additionally, we calculate the number of entanglements between individual CNTs from representative SEM images. In the case of S1–S2 we measure 3.5 ± 1.1 contact points/μm^2^, and for S3–S4, 6.9 ± 1.7 contact points/μm^2^.

Differences in surface properties and composition of the steel substrates can explain the differences in CNT diameters and density for the various substrates. As can be seen from Table 1, steels S3–S4 are richer in iron alloys like Fe-Ni and Fe-Cr, compared to S1–S2. In literature, excess of Cr and Ni over Fe, has been associated with the formation of CNTs with smaller diameter and narrower distribution, which is in agreement with our observations of CNTs with smaller diameters in samples S3–S4 [55,56]. Additionally, samples S3 and S4 have a higher surface roughness and a higher surface energy compared to S1 and S2. The higher surface roughness combined with better wettability can inhibit catalyst particle mobility and thus hinter particle coalescence [57,58]. Therefore, on S3–S4 the available catalyst size would be smaller, thus leading to smaller CNT diameters, which is the result of our experimental observation [58,59]. The larger CNT diameter and broader distribution found on samples S1–S2 can be justified by the significantly lower Cr and Ni content, as well as the smoother surface and lower surface energy of these steels. Lower surface roughness and energy lead to catalyst particles with higher mobility, which would allow larger clustering, and thus, the SEM-observed CNTs with larger diameters can be justified.

To evaluate the possible differences between CNT forests grown on Si wafers versus steel substrates, we perform control experiments. From SEM images of CNTs grown on silicon, presented in Supplementary Material (Figure S1a,b), the measured forest height is 1.0 ± 0.05 mm, the CNT diameter is 70 ± 10 nm, and there are 11 ± 2 contact points/μm^2^. The average density is 0.21 ± 0.08 g/cm^3^ and growth rate is 20 µm/min. These morphological features are in agreement with previous reports on CNT forests grown via the floating catalyst or fixed catalyst processes [40,60,61]. The higher growth rate and density of the forests grown on Si can be justified by the substrate composition. In the case of Si wafer substrate, ferrocene feedstock is the only source of catalyst particles, whereas on the steel substrates, since the surface is rich in metals such as Fe, Cr, Ni, Mo, and Cu, the corresponding metal oxides are formed during annealing at elevated temperatures in air flow, serving as additional catalyst sites [11,14,29]. In Si substrates, the dissociated carbon atoms are diffusing over Fe catalyst particles, while on steel substrates there are additional sites available composed primarily of Cr and Ni. Carbon solubility in Cr and Ni is lower compared to Fe [55,62]. As such, the lower growth rate, and subsequently the lower density, in steel substrates can be justified by the presence of Cr and Ni, in agreement with previous reports from Chen et al. [55].

**Figure 3:**
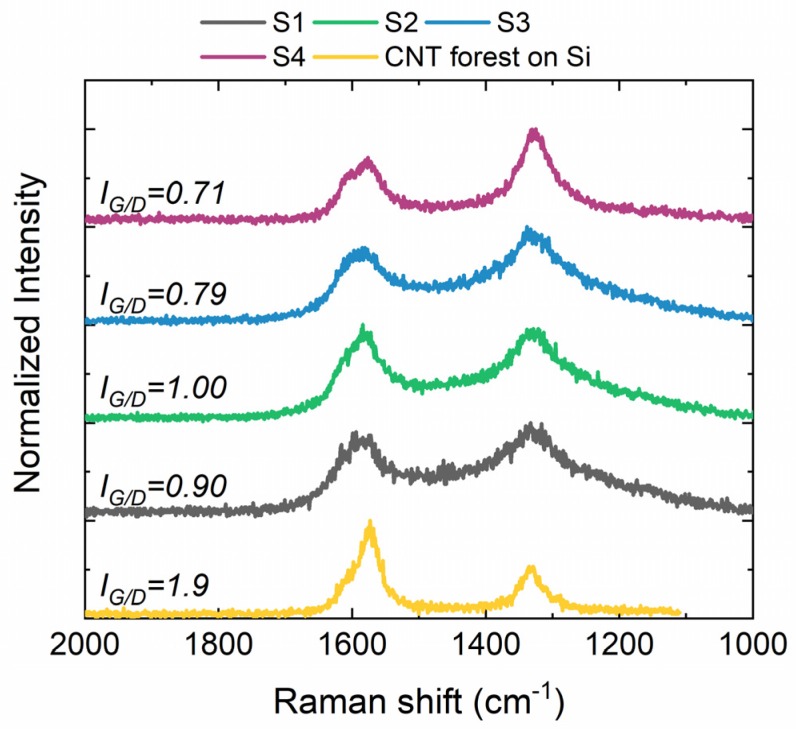
Raman spectra obtained from the CNT forests grown on the different substrates. The *I*_*G*/*D*_ ratio is indicated for every substrate type.

To analyze the quality of the produced CNTs, we perform Raman spectroscopy measurements (Figure 3). The Raman peak at around 1600 cm^−1^ (G band) is associated to the graphitic carbon, while the one around 1340 cm^−1^ (D band) is related to a hybridized vibrational mode arising from defects on the graphene structure. The ratio between G and D peaks (*I*_*G*/*D*_) can be used as an indication of the CNTs’ quality [52]. CNT forests grown on Si wafers result in a high *I*_*G*/*D*_ ratio of 1.9, while the CNTs grown on the steel substrates range from 0.7 to 1.0, indicating a lower defect density for the Si-grown CNTs. The small variation between samples grown on S1–S2 and S3–S4, with the former presenting higher values (0.9–1.0) compared to the latter (0.71–0.79), suggest a lower defect density and higher degree of alignment for forests grown on S1–S2 in comparison to those grown on S3–S4 steels [52,63].

### 3.2. Quasi-Static Characterization

**Figure 4:**
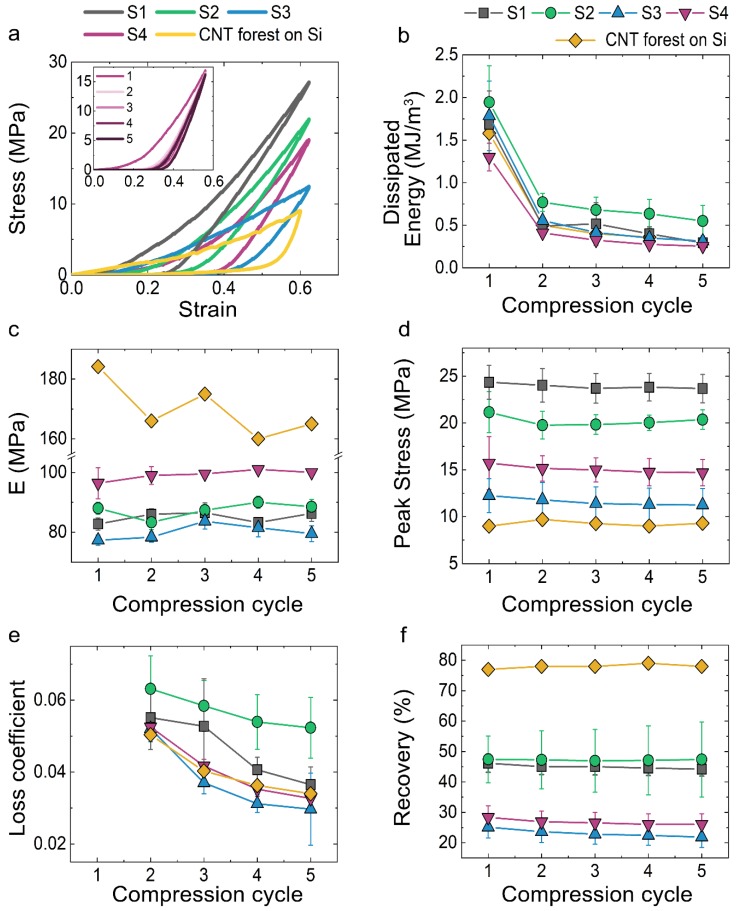
(**a**) Stress-strain curves for CNT forests grown on all substrates; five consecutive compression cycle stress-strain data for S4 (inset); (**b**) dissipated energy; (**c**) unloading modulus; (**d**) peak stress; (**e**) loss coefficient; and (**f**) recovery percentage for CNT forests grown on all substrates; (**b**–**f**) account for five consecutive compression cycles on each sample.

We study the mechanical performance of the CNT forests subjected to quasi-static compressions for five consecutive cycles at a stain rate of 0.02 s^−1^. Results indicate that after the third compression cycle, the forests reach a steady-state response (data for the first five compression cycles for S4 presented in the inset of Figure 4a). Representative stress-strain curves for the first compression cycle for the different types of steel substrates, as well as for the CNT forest grown on Si wafer, are presented in Figure 4a. From the obtained curves, we extract the dissipated energy (Figure 4b), unloading modulus (Figure 4c), peak stress (Figure 4d), loss coefficient (Figure 4e), and recovery (Figure 4f), for each of the five consecutive compression cycles.

At the first compression cycle, samples S1, S2, and S3 have similar dissipated energy values, in the range of 1.7–1.9 MJ/m^3^. The CNT forest on S4 dissipates distinctly less energy, with an average value of 1.3 MJ/m^3^. CNTs grown on Si dissipate 1.9 MJ/m^3^, confirming that the energy dissipation is at the same order of magnitude for all CNT forests grown on Si and on steel. For the subsequent cycles, we note a decrease in the amount of dissipated energy, which levels off at about 20–30% of the original value on all substrates. The same behavior is observed for the CNTs grown on Si wafer. The lower dissipated energy of S4, compared to the rest of steel samples and CNTs grown on Si, can be attributed to the lower CNT density of that sample, as demonstrated by Raney et al. and Pathak et al. [6,10].

The unloading modulus obtained for all samples ranges between 75 MPa and 100 MPa, with S4 presenting the highest stiffness, followed by S2, S1, and S3. In all steel samples, the unloading modulus is almost constant, with deviations within ±10% for all the successive compression cycles. For CNTs grown on Si, the modulus is found to be higher and constant throughout cyclic loading, averaging at 170 MPa for all cycles. The higher stiffness found on CNTs grown on Si compared to steels can be correlated to the notably higher density, the higher number of entanglements between individual tubes, as well as to the detected lower defect density of the CNT forests grown on Si. The higher number of inter-tube contacts in S4 compared to the rest of the steel samples, can cause stronger inter-tube interactions [64] in the former case, which together with the lower CNT density can justify the higher stiffness of S4 [6,65].

The peak stress remains almost constant through all the consecutive compressions on all samples, with variations less than ±10%. Specifically, the CNTs on the S1 steel present the highest peak stress value, peaking at 24.4 MPa, while for S2 the maximum reached stress is 21.2 MPa. For S4 and S3 steels, lower stress levels are observed for the first compression cycle reaching 15.7 and 12.3 MPa, respectively. The peak stress values for the CNTs grown on Si are also constant, with an average at 10 MPa.

The loss coefficient, which reflects the degree to which a material dissipates energy, decreases slightly for each consecutive cycle for all steel types. We obtain similar values for all samples grown on steels and on Si, which lie between 2 × 10^−2^ and 7 × 10^−2^. We also investigate the deformation recovery of the CNTs after compression. We find that CNTs grown on the steels S1 and S2 show the largest recovery, averaging at 45% for all compressive cycles. The CNTs on S3 and S4 also have an almost constant recovery throughout all cycles, at 23% and 26%, respectively. CNT forests grown on Si recover significantly more, with an average at 78% throughout all compression cycles. The CNT recovery has been associated with sample density, which can also be seen through our observations [6,41]. Samples S3–S4 with lower density have lower resilience, recovering distinctly less after their compression, compared to the denser S1–S2 samples.

Overall, our results indicate that the denser, thicker, and less interconnected CNT forests grown on S1–S2 exhibit the highest energy dissipation, peak stress, loss coefficient, and recovery, while the less dense, smaller diameter, and more entangled CNTs grown on sample S4, result in higher stiffness. This is in agreement with previous observations of CNTs grown on Si [10,41].

**Figure 5:**
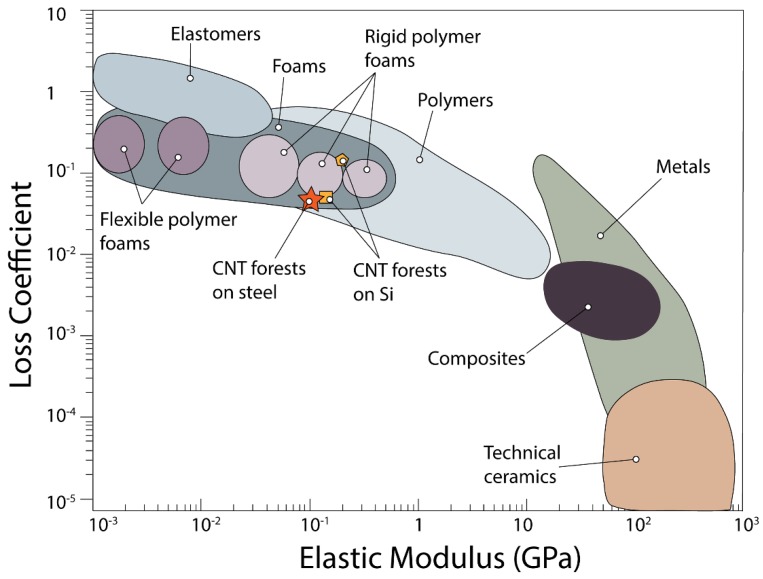
Loss coefficient versus elastic modulus for different material types. The performance of CNT forests on steel is marked by the star symbol, square marker represents CNT forests grown on Si with the same method, and pentagon marker represents CNTs grown on Si through a different method. Data from our work and literature-reported values [10,39].

The mechanical performance of the CNT forests suggests that they may be suitable for vibration mitigation and protective applications because they combine high energy dissipation, strength, and recoverability. We compare the performance of our materials with other CNT foams or polymeric foams of similar density, which are used in those applications, in an Ashby plot [46] (Figure 5). The unloading modulus and loss coefficient of CNT forests grown on metal substrates is comparable to that of rigid polymer foams such as phenolic foams (densities in the range of 0.3–2.0 g/cm^3^) [47]. However, the rigid polymer foams fail catastrophically upon compression, while CNT forests offer the same performance coupled with recoverability. Flexible polymer foams on the other hand, which lie on the far left of the property plot presented in Figure 5, offer the advantage of recoverability but have considerably lower stiffness (10^−3^–10^−2^ GPa) compared to CNTs. Therefore, the mechanical performance of CNT forests combines advantages otherwise not found in one group of foams. The Ashby plot of Figure 4 also offers a comparison with literature-reported mechanical performance of CNT forests. Forests grown on Si fabricated with the floating catalyst method and tested under similar conditions are depicted with the orange square symbol [10], and CNT forests fabricated with a different method are represented by the orange pentagon symbol [39]. The comparison confirms our observation that CNT forests grown on steels have similar mechanical properties to CNTs grown on Si.

### 3.3. Wetting Properties

Corrosion protection on steel surfaces can be obtained using superhydrophobic coatings which prevent water diffusion to the surfaces, thereby extending their service life [34,35,66]. CNTs have been suggested as alternatives because they combine superhydrophobicity with thermal and electrical conductivity, which their organic counterparts lack [34,35,67]. To characterize the hydrophobicity of our coatings, we perform water contact angle measurements. The results are summarized in Table 2.

All the fabricated CNT forests are hydrophobic, and in some cases superhydrophobic. The best performance is obtained for the CNT forest on S2, which has a water contact angle of 161.5°, followed by S3, S1, and S4, for which the contact angles vary from 153.6 to 144.8°. The measured contact angle of the control CNT sample grown on a Si wafer is 133.2 ± 5.8°, which is the lowest measured value. This can be explained by the enhanced surface roughness of the steel surfaces, compared to that of a Si wafer. The higher surface roughness of steel yields a less uniform CNT height, therefore a rougher top surface of the forest. It is known that a higher surface roughness leads to higher contact angles [68,69], and therefore this can count for the higher contact angle of CNT forests on steel versus Si.

The morphological differences of the CNTs on the steel substrates can justify the lower hydrophobicity of S4 compared to S1, S2, and S3. As previously mentioned, the CNTs in S4 are more entangled, which could lead to more non-aligned CNT tips exposed on the surface and which can in turn explain the lowest hydrophobicity compared to the rest of the samples. The samples S1–S2 have lower CNT entanglement, suggesting more aligned CNT tips on the surface and thus a higher hydrophobicity can be expected.

### 3.4. Adhesion Tests

**Figure 6:**
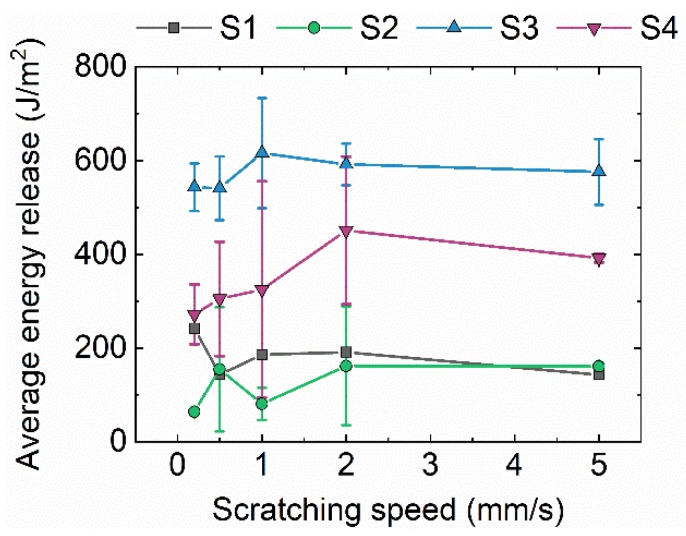
Calculated energy release as a function of the scratching speed, for the different types of samples.

To evaluate the adhesion between the CNT forests and the steel substrates, we use a custom-made scratching setup (Figure 1a). As described previously, the force during scratching along the length of the substrate is continuously monitored. At the time point in which the razor blade scratches the CNT forest a distinct force increase is recorded, as shown Figure 1b, in a representative example of an obtained force-time curve, corresponding to sample S3. Using Equation (8) we calculate the average energy released during scratching for each substrate. The results for all samples, considered at five different scratching speeds, show that the CNTs adhere more efficiently on the surface of S3–S4, compared to S1–S2 at every tested scratch speed (Figure 6). Specifically, for S3 the average energy release values are in the range of 530–620 J/m^2^, while for S4 they are within 250–450 J/m^2^. For CNTs on S1–S2 the adhesion is almost the same with energy release values around 100–200 J/m^2^.

As previously discussed and shown in Table 1, the surface energy of steel substrates is highest for S3, and then in descending order for S4, S1, and S2, respectively. Additionally, S3–S4 have larger surface roughness compared to S1–S2. The combination of higher surface energy and roughness, found in S4 and S3 can explain the better adhesion that CNTs have on these surfaces, compared to S1–S2.

Due to the use of different techniques to measure adhesion-related strength or energy release, a comparison with all literature reported values is not always possible [12,14,52,53]. The results which could be compared with our measurements are those reported by Cao et al. [14], Ageev et al. [53], and Lahiri et al. [52]. Cao et al. grew CNT forests on the end of a silicon carbide (SiC) micron-thick fiber in the form of a brush. They determined the adhesion of the CNTs on SiC by taping over the CNT area and pulling them away from their substrate with a tension machine. The calculated energy based on their results, assuming a pulling length equal to the total length of the bristle in their brush, is 84 J/m^2^ for CNTs grown on SiC fibers without post-growth treatment [14]. Ageev et al. used an AFM to measure the adhesion of CNT forests grown through plasma-enhanced chemical vapor deposition (PECVD) on a Si wafer on a Ti/Ni layer (buffer and catalyst respectively) and reported an adhesion strength of 0.55–1.9 mJ/m^2^ [53]. Finally, Lahiri et al., using a nanoindenter, scratched through un-oriented CNTs grown on Si and Cu wafers and determined an adhesion of ~80–400 J/m^2^ for Si and 600–1070 J/m^2^ for Cu wafers [52]. The results from Lahiri et al. for Si wafer-grown CNTs are comparable to the energy release values found in our work, while those for Cu-grown CNTs indicate a higher adhesion than the one found in our samples. However, it should be noted that in the case of randomly oriented CNTs the contact area between the individual tube and the substrate can be much higher than in the case of vertically-aligned CNTs where mostly only the base of each tube is in contact with the substrate. Therefore, the calculated energy release per unit area can be an overestimation in the case of un-oriented CNTs. The results of Cao et al. are lower than the values measured in the present work, which can be attributed to the different type and geometry of the substrates used in that work. Finally, the results from Ageev et al. are significantly lower than the rest of the reported values, possibly due to the fundamentally different measurement method employed in that case.

## 4. Conclusions

In this work, we report the synthesis, structural, and mechanical characterization of CNT forests grown on commercial steel substrates. The CNT forests are grown using the floating catalyst method, directly on top of the steel substrates without the use of intermediate adhesive layers, to allow a robust direct contact. The results reveal that CNT forests grown on steel combine high energy dissipation, high compressive strength, and stiffness with recoverability. Their mechanical performance, lightweight, and superhydrophobicity, combined with their adhesion to steel, highlight the possibility of using carbon nanotube forests as a protective multifunctional layer material for metal surfaces. Our results show that the steel substrate composition and morphology determine the CNT diameter and density, which govern their mechanical performance. CNTs with larger diameter and density are less interconnected and have the highest energy dissipation, peak stress, loss coefficient, and recovery, as well as higher hydrophobicity. CNTs with smaller diameter, density, and higher amount of inter-tube entanglements result in higher stiffness and lower hydrophobicity.

## Figures and Tables

**Table 1:** Stainless steel substrates nominal compositions and surface characteristics.

Steel sample	Cr (wt%)	Ni (wt%)	Mo (wt%)	Cu (wt%)	C (wt%)	Surface roughness (µm)	Surface energy, *γ_s_* (mJ/m^2^)
S1	1.9–2.6			<0.3	0.05–0.15	0.2	35.4
S2	8–9.5	<0.4			0.08–0.12	0.13	30.7
S3	11–12.5	2.2–3	1.3–1.9		0.1–0.14	0.36	53.7
S4	13.5–16	4–6	1.2–2	1–2	0.07	0.85	44.8

**Table 2:** Contact angle between a water droplet and the CNT forests grown on different substrates.

Substrate Used	Average Water Contact Angle (Degrees)
S1	150.6 ± 10.8
S2	161.5 ± 4.4
S3	153.6 ± 5.8
S4	144.8 ± 0.3
Si	133.2 ± 5.8

## Supplementary Materials

The following are available online at https://www.mdpi.com/2079-4991/9/3/444/s1, Figure S1: SEM images of CNTs grown on silicon.

## References

[1] Cao A., Dickrell P.L., Sawyer W.G., Ghasemi-Nejhad M.N., Ajayan P.M. (2005). Super-Compressible Foamlike Carbon Nanotube Films. Science.

[2] Qu L., Dai L., Stone M., Xia Z., Wang Z.L. (2008). Carbon Nanotube Arrays with Strong Shear Binding-On and Easy Normal Lifting-Off. Science.

[3] De Volder M.F.L., Tawfick S.H., Baughman R.H., Hart A.J. (2014). Carbon Nanotubes: Present and Future Commercial Applications. Science.

[4] Schnorr J.M., Swager T.M. (2011). Emerging Applications of Carbon Nanotubes. Chem. Mater..

[5] Thevamaran R., Meshot E.R., Daraio C. (2015). Shock Formation and Rate Effects in Impacted Carbon Nanotube Foams. Carbon.

[6] Raney J.R., Misra A., Daraio C. (2011). Tailoring the Microstructure and Mechanical Properties of Arrays of Aligned Multiwall Carbon Nanotubes by Utilizing Different Hydrogen Concentrations during Synthesis. Carbon.

[7] Thevamaran R., Karakaya M., Meshot E.R., Fischer A., Podila R., Rao A.M., Daraio C. (2015). Anomalous Impact and Strain Responses in Helical Carbon Nanotube Foams. RSC Adv..

[8] Pour Shahid Saeed Abadi P., Hutchens S.B., Greer J.R., Cola B.A., Graham S. (2012). Effects of Morphology on the Micro-Compression Response of Carbon Nanotube Forests. Nanoscale.

[9] Yaglioglu O., Cao A., Hart A.J., Martens R., Slocum A.H. (2012). Wide Range Control of Microstructure and Mechanical Properties of Carbon Nanotube Forests: A Comparison between Fixed and Floating Catalyst CVD Techniques. Adv. Funct. Mater..

[10] Pathak S., Raney J.R., Daraio C. (2013). Effect of Morphology on the Strain Recovery of Vertically Aligned Carbon Nanotube Arrays: An in Situ Study. Carbon.

[11] Vander Wal R.L., Hall L.J. (2003). Carbon Nanotube Synthesis upon Stainless Steel Meshes. Carbon.

[12] Talapatra S., Kar S., Pal S.K., Vajtai R., Ci L., Victor P., Shaijumon M.M., Kaur S., Nalamasu O., Ajayan P.M. (2006). Direct Growth of Aligned Carbon Nanotubes on Bulk Metals. Nat. Nano.

[13] Karwa M., Iqbal Z., Mitra S. (2006). Scaled-up Self-Assembly of Carbon Nanotubes inside Long Stainless Steel Tubing. Carbon.

[14] Cao A., Veedu V.P., Xuesong L.I., Yao Z., Ghasemi-Nejhad M.N., Ajayan P.M. (2005). Multifunctional Brushes Made from Carbon Nanotubes. Nat. Mater..

[15] Masarapu C., Wei B. (2007). Direct Growth of Aligned Multiwalled Carbon Nanotubes on Treated Stainless Steel Substrates. Langmuir.

[16] Sano N., Hori Y., Yamamoto S., Tamon H. (2012). A Simple Oxidation-Reduction Process for the Activation of a Stainless Steel Surface to Synthesize Multi-Walled Carbon Nanotubes and Its Application to Phenol Degradation in Water. Carbon.

[17] Hordy N., Mendoza-Gonzalez N.Y., Coulombe S., Meunier J.L. (2013). The Effect of Carbon Input on the Morphology and Attachment of Carbon Nanotubes Grown Directly from Stainless Steel. Carbon.

[18] Sano N., Yamamoto S., Tamon H. (2014). Cr as a Key Factor for Direct Synthesis of Multi-Walled Carbon Nanotubes on Industrial Alloys. Chem. Eng. J..

[19] Zhuo C., Wang X., Nowak W., Levendis Y.A. (2014). Oxidative Heat Treatment of 316L Stainless Steel for Effective Catalytic Growth of Carbon Nanotubes. Appl. Surf. Sci..

[20] Romero P., Oro R., Campos M., Torralba J.M., De Villoria R.G. (2015). Simultaneous Synthesis of Vertically Aligned Carbon Nanotubes and Amorphous Carbon Thin Films on Stainless Steel. Carbon.

[21] Reinhardt H., Hellmann C., Nürnberger P., Kachel S., Hampp N. (2017). Free Form Growth of Carbon Nanotube Microarchitectures on Stainless Steel Controlled via Laser-Stimulated Catalyst Formation. Adv. Mater. Interfaces.

[22] Camilli L., Scarselli M., Del Gobbo S., Castrucci P., Nanni F., Gautron E., Lefrant S., De Crescenzi M. (2011). The Synthesis and Characterization of Carbon Nanotubes Grown by Chemical Vapor Deposition Using a Stainless Steel Catalyst. Carbon.

[23] Hashempour M., Vicenzo A., Zhao F., Bestetti M. (2013). Direct Growth of MWCNTs on 316 Stainless Steel by Chemical Vapor Deposition: Effect of Surface Nano-Features on CNT Growth and Structure. Carbon.

[24] Pattinson S.W., Viswanath B., Zakharov D.N., Li J., Stach E.A., Hart A.J. (2015). Mechanism and Enhanced Yield of Carbon Nanotube Growth on Stainless Steel by Oxygen-Induced Surface Reconstruction. Chem. Mater..

[25] Pakdee U., Chiangga S., Suwannatus S., Limsuwan P. (2017). Growth of MWCNTs on Flexible Stainless Steels without Additional Catalysts. J. Nanomater..

[26] Raja Noor Amalina Raja S., Mohd Asyadi A., Mohd Ambri M., Seman R.N.A.R., Azam M.A., Mohamed M.A. (2016). Highly Efficient Growth of Vertically Aligned Carbon Nanotubes on Fe–Ni Based Metal Alloy Foils for Supercapacitors. Adv. Nat. Sci. Nanosci. Nanotechnol..

[27] Li D., Cheng Y., Wang Y., Zhang H., Dong C., Li D. (2016). Improved Field Emission Properties of Carbon Nanotubes Grown on Stainless Steel Substrate and Its Application in Ionization Gauge. Appl. Surf. Sci..

[28] Park D., Kim Y.H., Lee J.K. (2003). Synthesis of Carbon Nanotubes on Metallic Substrates by a Sequential Combination of PECVD and Thermal CVD. Carbon.

[29] Park S.J., Lee D.G. (2006). Development of CNT-Metal-Filters by Direct Growth of Carbon Nanotubes. Curr. Appl. Phys..

[30] Camilli L., Scarselli M., Del Gobbo S., Castrucci P., Lamastra F.R., Nanni F., Gautron E., Lefrant S., D’Orazio F., Lucari F. (2012). High Coercivity of Iron-Filled Carbon Nanotubes Synthesized on Austenitic Stainless Steel. Carbon.

[31] Kruehong S., Kruehong C., Artnaseaw A. (2016). Branched Carbon Fibres and Other Carbon Nanomaterials Grown Directly from 304 Stainless Steel Using a Chemical Vapour Deposition Process. Diam. Relat. Mater..

[32] Weiwei Z., Yu Z., Ningsheng X., Yuanming T., Runze Z., Yan S., Zhi X., Xuedong B., Jun C., Juncong S. (2017). Epitaxial Growth of Multiwall Carbon Nanotube from Stainless Steel Substrate and Effect on Electrical Conduction and Field Emission. Nanotechnology.

[33] Ashraf A., Salih H., Nam S., Dastgheib S.A. (2016). Robust Carbon Nanotube Membranes Directly Grown on Hastelloy Substrates and Their Potential Application for Membrane Distillation. Carbon.

[34] Sethi S., Dhinojwala A. (2009). Superhydrophobic Conductive Carbon Nanotube Coatings for Steel. Langmuir.

[35] Francesco De N., Paola C., Manuela S., Francesca N., Ilaria C., Maurizio De C., De Nicola F., Castrucci P., Scarselli M., Nanni F. (2015). Super-Hydrophobic Multi-Walled Carbon Nanotube Coatings for Stainless Steel. Nanotechnology.

[36] Suárez-Martínez R., Ocampo-Macias T., Lara-Romero J., Lemus-Ruiz J., Jiménez-Alemán O., Chiñas-Castillo F., Sagaro-Zamora R., Jiménez-Sandoval S., Paraguay-Delgado F. (2016). Synthesis and Tribological Performance of Carbon Nanostructures Formed on AISI 316 Stainless Steel Substrates. Tribol. Lett..

[37] Baddour C.E., Fadlallah F., Nasuhoglu D., Mitra R., Vandsburger L., Meunier J.L. (2009). A Simple Thermal CVD Method for Carbon Nanotube Synthesis on Stainless Steel 304 without the Addition of an External Catalyst. Carbon.

[38] Abdulrahaman M.A., Abubakre O.K., Abdulkareem S.A., Tijani J.O., Aliyu A., Afolabi A.S. (2017). Effect of Coating Mild Steel with CNTs on Its Mechanical Properties and Corrosion Behaviour in Acidic Medium. Adv. Nat. Sci. Nanosci. Nanotechnol..

[39] Pathak S., Lim E.J., Pour Shahid Saeed Abadi P., Graham S., Cola B.A., Greer J.R. (2012). Higher Recovery and Better Energy Dissipation at Faster Strain Rates in Carbon Nanotube Bundles: An in-Situ Study. ACS Nano.

[40] Misra A., Raney J.R., De Nardo L., Craig A.E., Daraio C. (2011). Synthesis and Characterization of Carbon Nanotube-Polymer Multilayer Structures. ACS Nano.

[41] Bradford P.D., Wang X., Zhao H., Zhu Y.T. (2011). Tuning the Compressive Mechanical Properties of Carbon Nanotube Foam. Carbon.

[42] Xu M., Futaba D.N., Yumura M., Hata K. (2012). Alignment Control of Carbon Nanotube Forest from Random to Nearly Perfectly Aligned by Utilizing the Crowding Effect. ACS Nano.

[43] Bedewy M., Meshot E.R., Reinker M.J., Hart A.J. (2011). Population Growth Dynamics of Carbon Nanotubes. ACS Nano.

[44] Roumeli E., Pavlidou E., Avgeropoulos A., Vourlias G., Bikiaris D.N.D., Chrissafis K. (2014). Factors Controlling the Enhanced Mechanical and Thermal Properties of Nanodiamond-Reinforced Cross-Linked High Density Polyethylene. J. Phys. Chem. B.

[45] Laurent C., Flahaut E., Peigney A. (2010). The Weight and Density of Carbon Nanotubes versus the Number of Walls and Diameter. Carbon.

[46] Felekis D., Vogler H., Mecja G., Muntwyler S., Nestorova A., Huang T., Sakar M.S., Grossniklaus U., Nelson B.J. (2015). Real-Time Automated Characterization of 3D Morphology and Mechanics of Developing Plant Cells. Int. J. Rob. Res..

[47] Ashby M.F. (2011). Material Property Charts. Materials Selection in Mechanical Design.

[48] Chapman B.N. (1974). Thin-Film Adhesion. J. Vac. Sci. Technol..

[49] Kleinbichler A., Pfeifenberger M.J., Zechner J., Moody N.R., Bahr D.F., Cordill M.J. (2017). New Insights into Nanoindentation-Based Adhesion Testing. JOM.

[50] Steinmann P.A., Hintermann H.E. (1985). Adhesion of TiC and Ti(C,N) Coatings on Steel. J. Vac. Sci. Technol. A.

[51] Akono A.T., Reis P.M., Ulm F.J. (2011). Scratching as a Fracture Process: From Butter to Steel. Phys. Rev. Lett..

[52] Lahiri I., Lahiri D., Jin S., Agarwal A., Choi W. (2011). Carbon Nanotubes: How Strong Is Their Bond with the Substrate?. ACS Nano.

[53] Ageev O.A., Blinov Y.F., Il’ina M.V., Il’in O.I., Smirnov V.A., Tsukanova O.G. (2016). Study of Adhesion of Vertically Aligned Carbon Nanotubes to a Substrate by Atomic-Force Microscopy. Phys. Solid State.

[54] Yu M., Funke H.H., Falconer J.L., Noble R.D. (2009). High Density, Vertically-Aligned Carbon Nanotube Membranes. Nano Lett..

[55] Chen G., Seki Y., Kimura H., Sakurai S., Yumura M., Hata K., Futaba D.N. (2014). Diameter Control of Single-Walled Carbon Nanotube Forests from 1.3-3.0 Nm by Arc Plasma Deposition. Sci. Rep..

[56] Kim N.S., Lee Y.T., Park J., Ryu H., Lee H.J., Choi S.Y., Choo J. (2002). Dependence of the Vertically Aligned Growth of Carbon Nanotubes on the Catalysts. J. Phys. Chem. B.

[57] De Los Arcos T., Garnier M.G., Seo J.W., Oelhafen P., Thommen V., Mathys D. (2004). The Influence of Catalyst Chemical State and Morphology on Carbon Nanotube Growth. J. Phys. Chem. B.

[58] Jourdain V., Bichara C. (2013). Current Understanding of the Growth of Carbon Nanotubes in Catalytic Chemical Vapour Deposition. Carbon.

[59] Gohier A., Ewels C.P., Minea T.M., Djouadi M.A. (2008). Carbon Nanotube Growth Mechanism Switches from Tip- to Base-Growth with Decreasing Catalyst Particle Size. Carbon.

[60] Nessim G.D., Hart A.J., Kim J.S., Acquaviva D., Oh J., Morgan C.D., Seita M., Leib J.S., Thompson C.V. (2008). Tuning of Vertically-Aligned Carbon Nanotube Diameter and Areal Density through Catalyst Pre-Treatment. Nano Lett..

[61] Raney J.R., Fraternali F., Daraio C. (2013). Rate-Independent Dissipation and Loading Direction Effects in Compressed Carbon Nanotube Arrays. Nanotechnology.

[62] Moisala A., Nasibulin A.G., Kauppinen E.I. (2003). The Role of Metal Nanoparticles in the Catalytic Production of Single-Walled Carbon Nanotubes—A Review. J. Phys. Condens. Matter.

[63] Meshot E.R., Zwissler D.W., Bui N., Kuykendall T.R., Wang C., Hexemer A., Wu K.J.J., Fornasiero F. (2017). Quantifying the Hierarchical Order in Self-Aligned Carbon Nanotubes from Atomic to Micrometer Scale. ACS Nano.

[64] Zhao J., Wang F., Zhang X., Liang L., Yang X., Li Q., Zhang X. (2018). Vibration Damping of Carbon Nanotube Assembly Materials. Adv. Eng. Mater..

[65] Won Y., Gao Y., Panzer M.A., Xiang R., Maruyama S., Kenny T.W., Cai W., Goodson K.E. (2013). Zipping, Entanglement, and the Elastic Modulus of Aligned Single-Walled Carbon Nanotube Films. Proc. Natl. Acad. Sci. USA.

[66] Fihri A., Bovero E., Al-Shahrani A., Al-Ghamdi A., Alabedi G. (2017). Recent Progress in Superhydrophobic Coatings Used for Steel Protection: A Review. Colloids Surfaces A Physicochem. Eng. Asp..

[67] Lau K.K.S., Bico J., Teo K.B.K., Chhowalla M., Amaratunga G.A.J., Milne W.I., McKinley G.H., Gleason K.K. (2003). Superhydrophobic Carbon Nanotube Forests. Nano Lett..

[68] Wenzel R.N. (1949). Surface Roughness and Contact Angle. J. Phys. Colloid Chem..

[69] Miwa M., Nakajima A., Fujishima A., Hashimoto K., Watanabe T. (2000). Effects of the Surface Roughness on Sliding Angles of Water Droplets on Superhydrophobic Surfaces. Langmuir.

