# Supplementary material for: Characterization of Vertically Aligned Carbon Nanotube Forests Grown on Stainless Steel Surfaces
Source: Nanomaterials (Basel). 2019 Mar 15;9(3):444. doi: 10.3390/nano9030444 (PMC6474093; doi:10.3390/nano9030444)
Supplement: Supplementary file 1 [file nanomaterials-09-00444-s001.pdf]

Supporting Information

Characterization of Vertically Aligned Carbon Nanotube Forests Grown on Stainless Steel Surfaces

Eleftheria Roumeli [1,2], Marianna Diamantopoulou [1], Marc Serra-Garcia [1,3], Paul Johanns [1], Giulio Parcianello [4] and Chiara Daraio [2,*]

[1] Department of Mechanical and Process Engineering, Swiss Federal Institute of Technology (ETH Zurich), 8092, Zurich, Switzerland; eroumeli@caltech.edu (E.R.); mdiamant@student.ethz.ch (M.D.); marc.serra.g@gmail.com (M.S.-G.); paul.johanns@epfl.ch (P.J.)

[2] Division of Engineering and Applied Science, California Institute of Technology, Pasadena, CA 91125, USA

[3] Department of Physics, Swiss Federal Institute of Technology (ETH Zurich), 8092, Zurich, Switzerland

[4] General Electric Switzerland, CH-5401 Baden, Switzerland; giulio.parcianello@gmail.com

[*] Correspondence: daraio@caltech.edu; Tel.: +1-626-395-8515

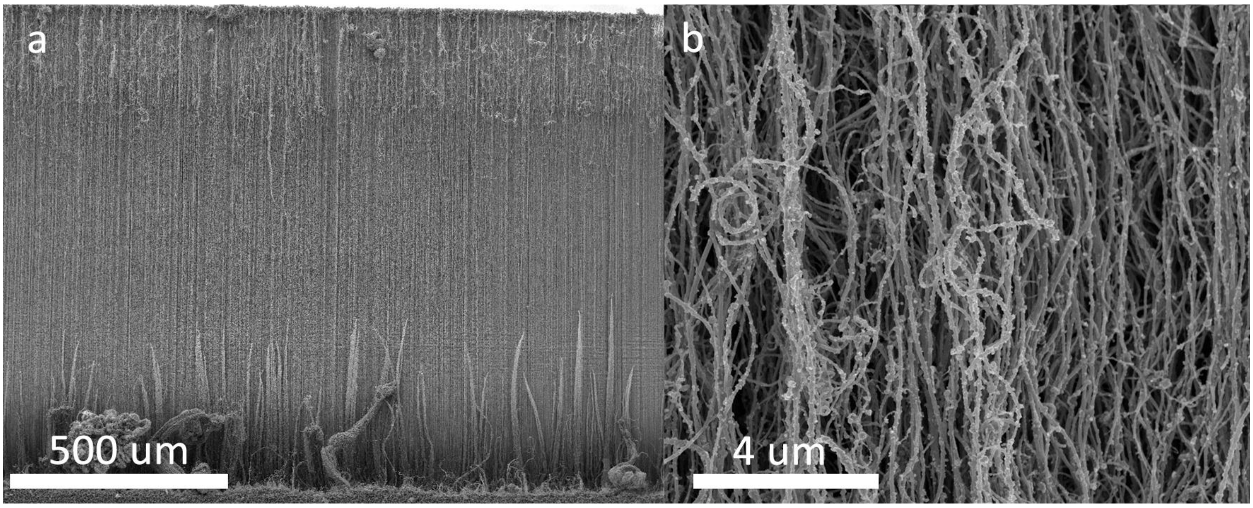

Figure 1. SEM images of CNT forest grown on silicon.
